# Burn Patients Infected With Metallo-Beta-Lactamase-Producing *Pseudomonas aeruginosa*: Multidrug-Resistant Strains

**DOI:** 10.5812/atr.18182

**Published:** 2014-06-01

**Authors:** Mojtaba Anvarinejad, Aziz Japoni, Noroddin Rafaatpour, Jalal Mardaneh, Pejman Abbasi, Maneli Amin Shahidi, Mohammad Ali Dehyadegari, Ebrahim Alipour

**Affiliations:** 1 Professor Alborzi Clinical Microbiology Research Center, Nemazee Hospital, Shiraz University of Medical Sciences, Shiraz, IR Iran

**Keywords:** Burn patients, *Pseudomonas aeruginosa*, Metallo-Beta-Lactamase, Drug Resistance

## Abstract

**Background:**

Metallo-beta-lactamase (MBL) producing *Pseudomonas aeruginosa* in the burn patients is a leading cause of morbidity and mortality and remains a serious health concern among the clinicians.

**Objectives:**

The aim of this study was to detect MBL-producing *P. aeruginosa* in burn patients and determine multidrug-resistant (MDR) strains, and respective resistance patterns.

**Patients and Methods:**

In this cross-sectional study, 270 strains of *P. aeruginosa* were isolated from the burn patients referred to Ghotbeddin Burn Hospital, Shiraz, Iran. Among them, 55 MBL-producing *P. aeruginosa* strains were isolated from 55 patients hospitalized in burn unit. Minimum inhibitory concentrations (MICs) and MBLs were determined by the E-test method.

**Results:**

Of the 55 burn cases, 29 (53%) were females and 26 (47%) males. Injured burn patients’ ages ranged from 16 to 87 years, with maximum number of cases in the age group of 16 to 36 years (n, 40; 72.7%). Overall, 32 cases were accidental (60%), and 22 were suicidal burns (40%). Of the 55 burn patients, 17 cases were expired (30%). All deaths were due to chemical exposures. In antibiotic susceptibility testing by E-test method, ceftazidime was the most effective one and 35 isolates (63.5%) were resistant to all the 11 tested antibiotics.

**Conclusions:**

Routine microbiological surveillance and careful in vitro testing of antibiotics prior to prescription and strict adherence to hospital antibiotic policy may help to prevent, treat, and control MDR and pandrug-resistant (PDR) *P. aeruginosa* strains in burn units.

## 1. Background

*Pseudomonas aeruginosa* is an obligate aerobic, motile, rod-shaped, Gram-negative bacterium, which is able to grow and survive in almost any environment and is resistant to temperature extremes (1). It is an opportunistic pathogen causing severe acute and chronic nosocomial infections, especially in immunocompromised hosts and patients with serious underlying medical conditions (2, 3). *Pseudomonas aeruginosa* generally exhibits intrinsic resistance against many antibiotics and is associated with a high mortality rate (4). Almost all the clinical cases of *P. aeruginosa* infections are associated with the compromised host defense as seen in burn patients (5). Bacterial infections following severe thermal injuries can be most simplistically attributed to the extensive breaches in the skin barrier (6). *P. aeruginosa* is a ubiquitous bacterium; hence, the risk of encounter this microorganism before the burns can heal is extremely high in sever burn patients (5, 7). Presently, more patients with burns die of pneumonia than of burn wound infection; however, burn wound sepsis remains an important infectious complication in such patients (8, 9). Various beta-lactam antibiotics, aminoglycosides, fluoroquinolones, and polymyxins have been used to treat burn patients infected with *P. aeruginosa* (10, 11). All the strains are prone to become resistant by mutations. Burn hospitals often harbor multidrug resistant *P. aeruginosa* (MDRPA) isolates that can serve as the source of infection for other patients hospitalized in burn ward (12).

Although systemic or topical antibiotic therapy has considerably improved the management of infectious diseases in the burn patients, many infections are not fully treated or eradicated by the application of antipseudomonal drugs and can, thus, become chronic infections (13, 14). For instance, burn patients can become colonized with antibiotic-resistant, nosocomial *P. aeruginosa* strains that are not easily eliminated by antibiotic therapy (15). Selection of an efficient antibiotic therapy regimen should be based on the ability of drug to inhibit the bacteria isolated from burn wound, periodic bacterial cultures, and monitoring the nosocomial infections in the burn wards (16). Outbreaks of cross-colonization and infection are a major challenge to the patients hospitalized in burn units (17). In almost all cases, the colonized patient is considered as a major reservoir for the epidemic strains (5, 17). It has been estimated that at least 50% of all deaths caused by burns are the result of infection, and untreatable infections have become a tragically frequent morbidity in patients infected with *P. aeruginosa* (18). Eradication of MDRPA from hospital burn wards is a demanding task; therefore, the detection of metallo-beta-lactamase (MBL) producing *P. aeruginosa* is necessary for controlling the spread of resistant strains as well as developing new therapeutic guidelines and prophylactic strategies to control the bacterial infection in patients with burn wounds.

## 2. Objectives

Given the existing limited data on MBL-producing *P. aeruginosa* strains in Shiraz, this study aimed to detect MBL-producing *P. aeruginosa* in burn patients, and determine the MDRPA strains in such isolates.

## 3. Patients and Methods

In this cross-sectional study, 270 strains of *P. aeruginosa* were isolated from the burn patients referred to Ghotbeddin Burn Hospital, Shiraz, Iran, during 2009-2010. Data concerning gender, age, duration of hospitalization, cause, site, degree, and types of burns (accidental or suicidal) were collected from the patients infected with MBL-producing *P. aeruginosa* through questionnaires filled by skilled nurses. This study was performed in accordance with the ethical standards laid down in the 1964 Helsinki declaration. All the patients were assigned code numbers. The design and protocol of the study were approved by the Ethics Committee of Professor Alborzi Clinical Microbiology Research Center (PACMRC), Shiraz, Iran.

### 3.1. Isolation and Identification of Metallo-Beta-Lactamase-Producing Pseudomonas aeruginosa

During a one-year period, 55 MBL-producing *P. aeruginosa* strains were isolated from 55 burn patients hospitalized in the burn unit. The isolation and identification of *P. aeruginosa* from wound specimens were performed by the following conventional procedures. Specimens were collected by sterile swabs after the removal of dressing and cleansing the wound surface by 70% alcohol. The burn samples were cultured on nutrient agar (NA; Oxoid Ltd, London, UK) and incubated at 35℃ to 37℃ overnight. Then any suspicious colony was subcultured and purified. The isolates were identified as *P. aeruginosa*, based on Gram staining, catalase test, oxidase test, triple sugar iron (TSI) fermentation, motility, color, pyocyanin pigment production, and odor. For final confirmation, biochemical tests embedded in the API-20E biochemical kit system (Bio-Merieux, France) and manual biochemical tests were used in accordance with the manufacturer’s instructions. Strains were preserved at -20℃ on tryptic soy broth (TSB; Oxoid Ltd, London, UK) containing 20% (v/v) glycerol. MBL E-test strips (AB BIODISK, Solna, Sweden) were used to screen class B beta-lactamase. Tests were performed and interpreted according to the manufacturer's instructions.

### 3.2. Antibiotic Susceptibility and Resistance Patterns

Minimum inhibitory concentrations (MICs) of the 11 antimicrobial agents routinely prescribed in burn centers against the 55 isolates of MBL-producing *P. aeruginosa* were determined by the E-test method (AB BIODISK, Solna, Sweden), as recommended by the National Committee For Clinical Laboratory Standard Institute (CLSI) (19). A bacterial suspension from growth in a tryptic soy agar (TSA) plate was prepared in 2 mL of Mueller-Hinton broth (MHB), and the turbidity was adjusted so that it was equivalent to that of a 0.5 McFarland standard. The bacterial suspension was streaked onto a 150-mm-diameter plate containing Mueller-Hinton agar (MHA); the plate was later incubated at 35 to 37℃ in ambient air for 16 to 18 hours. The MIC was read on the basis of the interception of the elliptical zone of growth inhibition with the graded E-test strip according to the manufacturer’s instructions. The antibiotics (Mast Co., UK) consisted of imipenem (10 μg), meropenem (10 μg), cefepime (30 μg), ceftazidime (30 μg), piperacillin/tazobactam (110 μg), ciprofloxacin (5 μg), tobramycin (10 μg), amikacin (30 μg), gentamicin (10 μg), ampicillin (10 μg), and aztreonam (30 μg). American typing collection (ATCC 27853) of *P. aeruginosa* was used as a control strain to determine antibacterial susceptibility.

## 4. Results

Among 270 strains, 55 MBL-producing *P. aeruginosa* strains were isolated from patients hospitalized in burn unit. Of the 55 burn cases, 29 (53%) were females and 26 (47%) males. the age of the burn patients ranged from 16 to 87 years, with maximum number of cases in the age group of 16 to 36 years (n = 40; 72.7%) (Table 1). Fourteen burn-injured patients (25.5%) were from urban areas and 41 (74.5%) were from rural. Thirty-three cases (60%) were accidental, and 22 (40%) were suicidal. Overall, 51 patients (93%) had chemical injuries, and 4 (7%) had electrical injuries. In contrast to males, burns due to chemical exposures were more frequent in females (52.7%). Of the 55 burn patients, 17 patients (30%) died. Females showed a high incidence of mortality (females, 70.5%; males, 29.5%; and female to male ratio, 2.4). All deaths were due to chemical exposures (Table 2). 

**Figure 1.**
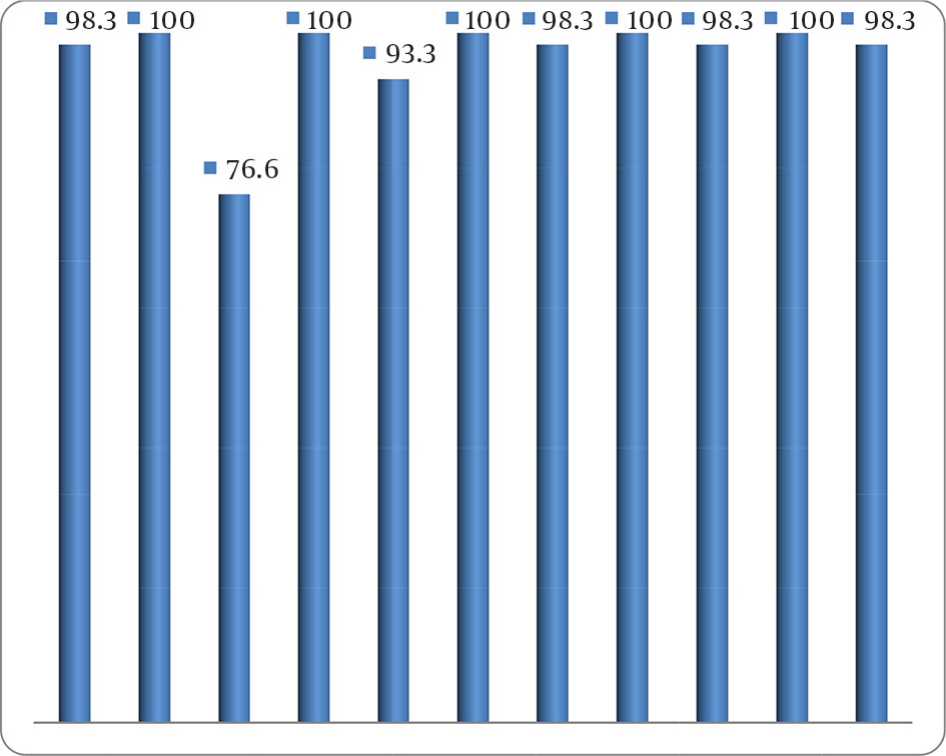
Antibiotic Resistance Profile in Metallo-Beta-Lactamase-Producing *Pseudomonas* *aeruginosa* Isolates (n = 55)

According to the in vitro antibiotic susceptibility testing by E-test method, ceftazidime was the most effective antibiotic (Figure 1). Thirty-five isolates (63.5%) were resistant to all the 11 tested antibiotics (Table 3).

**Table 1.** Demographic Features of the Burn Patients ^a^

Category	Results
**Age, y**	35 ± 17
16-36	40 (72.7)
37-56	9 (16.3)
> 56	6 (11)
**Residence**	
Rural	41 (74.5)
Urban	14 (25.5)
**Hospitalization, d**	25 ± 12
1-30	34 (61.8)
31-60	15 (27.2)
61-90	5 (9)
91-120	1 (1)

^a^ Data are presented as Mean ± SD or No. (%).

**Table 2.** Distribution of the Burn Patients in Terms of the Gender, Burn Cause, and Exposure Types

	Results
**Gender**	
Male (n = 26)	8 urban
	18 rural
Female (n = 29)	6 urban
	23 rural
**Burn cause**	
Accidental (n = 33)	23 males
	10 females
Suicidal (n = 22)	3 males
	19 females
**Exposure**	
Electricity (n = 4)	0 male
	4 females
Chemical (n = 51)	22 males
	29 females
Death (n = 17)	whole body except head (n = 7)
	whole body (n = 9)
	Legs (n = 1)

**Table 3.** Antibiotic Resistance Patterns of Metallo-Beta-Lactamase-Producing *Pseudomonas aeruginosa* Strains Isolated From Burn Patients ^a^^, ^^b^

Antibiotic Resistance Patterns	Frequency (n = 55)
**IMI, MEM, CAZ, ATM, TN, GM, PTZ, CPM, AK, CIP, AP**	35 (63.6)
**IMI, MEM, ATM, TN, GM, PTZ, CPM, AK, CIP, AP**	8 (14.5)
**IMI, MEM, ATM, TN, GM, PTZ, CPM, AK, AP**	5 (9.1)
**IMI, MEM, CAZ, ATM, TN, PTZ, CPM, AK, CIP, AP**	1 (1.8)
**MEM, CAZ, ATM, TN, GM, PTZ, CPM, AK, CIP**	1 (1.8)
**IMI, MEM, CAZ, ATM, PTZ, CPM, AP**	1 (1.8)
**IMI, MEM, CAZ, ATM, PTZ, CPM, AK, AP**	1 (1.8)
**IMI, CAZ, ATM, TN, GM, CPM, AK, AP**	1 (1.8)
**IMI, MEM, AK, AP**	1 (1.8)
**IMI, AK, CIP, AP**	1 (1.8)

^a^Abbreviations: AK, amikacin; AP, ampicillin; ATM, aztreonam; CAZ, ceftazidime; CIP, ciprofloxacin; CPM, cefepime; GM, gentamicin; IMI, imipenem; MEM, meropenem; PTZ, piperacillin/tazobactam; TN, tobramycin.

^b^Data are presented as No. (%).

## 5. Discussion

*P. aeruginosa* is an opportunistic pathogen, which is commonly associated with nosocomial infections (20). Antimicrobial resistance during therapy occurs frequently among the initially susceptible *P. aeruginosa* isolates, resulting in the emergence of resistance to multiple antibiotics (21). MBL-producing *P. aeruginosa* strains have been identified from clinical isolates worldwide with increasing frequency over the past several years, and these isolates are responsible for protracted nosocomial infections in different countries (22). The rising frequency of MDRPA strains is a matter of concern as effective antimicrobial options are significantly limited (23). In this study, females were affected more than males (53% vs. 47%). This may be due to more frequent involvement of females in household chores, which demand more exposure to fire (e.g. in cooking and heating). The results indicated that the mortality in our burn cases was 30%, which is lower than the rates in some other reports (24) but higher than the rates in most of other countries (25). The total body surface area burned (whole body in 50% and whole body except head in 41% of cases) was the most frequent factor related to mortality. The increasing severity of injuries caused by more skin loss, exposes the largest burns to more complications, thus, rising mortality rate. Another prominent finding in the present study was the high level of antibacterial resistance profile detected in the isolates of *P. aeruginosa*. Resistance to aztreonam, piperacillin/tazobactam, meropenem, imipenem, and ampicillin was seen in all the isolates. Therefore, when administering empirical treatment in burn patients with nosocomial infections with *P. aeruginosa*, MBL should be considered in case the patients are not responsive to carbapenem therapy. The level of MBL production among MDRPA strains seems to be greater than that estimated in Iran. Reports on MBL-producing *P. aeruginosa* isolates are increasing globally due to the increased beta-lactam usage and emergence of resistant bacteria under antibiotic pressure. Currently, CLSI document has no guidelines for detecting MBLs in *P. aeruginosa*. This alarming antibiotic resistance trend was seen for *P. aeruginosa* strains, as seen in previous studies (26). A similar report of MDRPA was also reported by other researchers. Although ceftazidime was found to be the most effective drug in the present study, resistance to this antibiotic was high (76.6%). In one study from Iran, higher resistance to ceftazidime was reported (27); this could be due to the reason that these are reserve drugs and are used as the last resort for MDRPA in hospital burn settings in Shiraz. In the current study, the bacteria isolated from only nine patients receiving empirical antibiotic therapy were sensitive to the prescribed antibiotics according to MIC method. The changes in the bacterial resistance patterns, as observed in the burn wards, could have important implications for both clinical settings and epidemiological purposes. Such a high antibiotic resistance in *P. aeruginosa* isolates is probably due to the selective pressure exerted on the bacteria because of the factors such as poor adherence to hospital antibiotic policy and excessive as well as indiscriminate use of broad-spectrum antimicrobial agents including beta-lactams, carbapenems, aminoglycosides, and quinolones. These MDRPA strains establish themselves in the hospital environment and, thereby, spread from one patient to another, from medical personnel to the patients, or among different units in the hospital. More recently, MDRPA and pandrug-resistant *P. aeruginosa* isolates have emerged in hospital burn units. Hence, there is hardly any effective antibiotic against pandrug-resistant strains, in which an outer membrane barrier of low permeability and an array of efficient multidrug efflux pumps are combined with multitudes of specific antibiotic resistance mechanisms. Treatment of the infections caused by these so-called “superbugs” remains challenging because the pool of effective antibiotics is shrinking and few new antipseudomonal antibiotics are in development. In burn patients, an effective and continuous surveillance for infection control and accordingly, regular tissue culturing for control purposes at least twice a week are recommended. Routine microbiological surveillance and careful in vitro testing of antibiotics prior to prescription and strict adherence to the hospital antibiotic policy may be helpful in the prevention, treatment, and control of MDRPA in the patients hospitalized in burn units. Further investigations should be done to determine the changing sensitivity profiles of the different gram-positive and Gram-negative bacteria and respective antimicrobial susceptibility patterns (28-30). In this regard, the hospitals should formulate an effective antibiotic policy. When the wound bacterial counts are more than 10^5^ microorganisms per gram of tissue, the risk of wound infection is great, skin graft survival is poor, and wound healing is delayed (31). Bacterial counts of less than 10^3^ organisms per gram, are not usually invasive and allow skin graft survival rates of more than 90%. The goals of the local wound management should be the prevention of viable tissues desiccation and the control of bacteria.
